# *DNMT1*, *DNMT3A* and *DNMT3B* gene variants in relation to ovarian cancer risk in the Polish population

**DOI:** 10.1007/s11033-013-2589-0

**Published:** 2013-05-12

**Authors:** Adrianna Mostowska, Stefan Sajdak, Piotr Pawlik, Margarita Lianeri, Paweł P. Jagodzinski

**Affiliations:** 1 Department of Biochemistry and Molecular Biology, Poznań University of Medical Sciences, 6 Święcickiego Street, 60-781 Poznan, Poland; 2 Clinic of Gynecological Surgery, Poznań University of Medical Sciences, Poznan, Poland

**Keywords:** Ovarian cancer, Polymorphism, *DNMT1*, *DNMT3A*, *DNMT3B*

## Abstract

Studies have demonstrated that changes in DNA methylation of cancer related genes can be an elementary process accounting for ovarian tumorigenesis. Therefore, we evaluated the possible association of single nucleotide polymorphisms (SNPs) of *DNA methyltransferases* (*DNMTs*) genes, including *DNMT1*, *DNMT3B*, and *DNMT3A*, with ovarian cancer development in the Polish population. Using PCR–RFLP and HRM analyses, we studied the prevalence of the *DNMT1* rs8101626, rs2228611 and rs759920, *DNMT3A* rs2289195, 7590760, rs13401241, rs749131 and rs1550117, and *DNMT3B* rs1569686, rs2424913 and rs2424932 SNPs in patients with ovarian cancer (*n* = 159) and controls (*n* = 180). The lowest p values of the trend test were observed for the *DNMT1* rs2228611 and rs759920 SNPs in patients with ovarian cancer (*p*_trend_ = 0.0118 and *p*_trend_ = 0.0173, respectively). Moreover, we observed, in the recessive inheritance model, that the *DNMT1* rs2228611 and rs759920 SNPs are associated with an increased risk of ovarian cancer development [OR 1.836 (1.143–2.949), *p* = 0.0114, *p*_corr_ = 0.0342, and OR 1.932 (1.185–3.152), *p* = 0.0078, *p*_cor=_0.0234, respectively]. However, none of other nine studied SNPs displayed significant contribution to the development of ovarian cancer. Furthermore, haplotype and multifactor dimensionality reduction analysis of the studied *DNMT1*, *DNMT3B*, and *DNMT3A* polymorphisms did not reveal either SNP combinations or gene interactions to be associated with the risk of ovarian cancer development. Our results may suggest that *DNMT1* variants may be risk factors of ovarian cancer.

**Electronic supplementary material:**

The online version of this article (doi:10.1007/s11033-013-2589-0) contains supplementary material, which is available to authorized users.

## Introduction

Ovarian cancer includes any malignant growth that may develop in disparate parts of the ovary; however, the majority of ovarian malignancies arise from the ovarian epithelium [1]. This type of cancer usually exhibits vague symptoms and is the one of the most lethal gynecological cancers in women in the United States and Europe [2]. Although ovarian cancer has been intensively studied, the underlying cause of this malignant disease remains undetermined [1, 3]. There is a lot of evidence that shows a reduced risk for ovarian cancer in women who use oral contraceptives, have greater parity, or breastfed long-term [3]. Known risk factors of ovarian cancer include infertility, early age of menarche, late age of menopause, inflammation, some environmental factors, and genetic background [3]. The known genetic factors encompass a few high-penetrance genes (e.g., *BRCA1*) and numerous moderate and low-penetrance ovarian cancer susceptibility genes [3]. However, these factors alone are not sufficient for ovarian tumorigenesis, which indicates that attention must be paid to the role of epigenetic status changes during ovarian carcinogenesis [4, 5].

The term epigenetics describes the reversible regulation of gene expression that does not occur due to changes in the DNA sequence [6, 7]. Epigenetic traits include DNA methylation, covalent modification of histones, and expression status of genes modulated by μRNA [6, 7].

DNA methylation in mammalian cells is typically restricted to covalent modification, in which a methyl group is added to a cytosine located in CpG dinucleotides in the genomic DNA [6]. CpG dinucleotides are frequently situated in rich CpG genomic sites designated as CpG islands, which are found in approximately half of all genes in humans [8]. Hypomethylation of regulatory DNA sequences increases the expression of proto-oncogenes and genes encoding proteins involved in genomic instability and malignant cell growth and metastasis [6, 9]. By contrast, hypermethylation of the promoter regions of tumor suppressor genes (TSGs) causes the transcriptional silencing of TSGs [6, 9].

There are three types of enzymatically active DNA methyltransferases (DNMTs), designated DNMT1, DNMT3A, and DNMT3B [6, 9]. Abnormal levels of DNMT1, DNMT3A and DNMT3B contributing to changes in the expression of cancer related genes have been found in different types of malignancies [10–13]. The DNMTs levels can be changed by single nuclear polymorphisms (SNPs) situated within their genes and which may affect the development of various cancers [14, 15]. Therefore, we selected 11 SNPs of *DNMT1*, *DNMT3A* and *DNMT3B* located in distinct blocks of linkage disequilibrium (LD) according to HapMap CEU data (http://hapmap.ncbi.nlm.nih.gov/) (Supplemental Table 1; Supplemental Fig. 1a–c). Furthermore, we aimed to study whether these *DNMT1*, *DNMT3A* and *DNMT3B* SNPs can be a genetic risk factor of ovarian cancer.

## Materials and methods

### Patients and controls

The patients include 159 women with histologically recognized ovarian carcinoma according to the International Federation of Gynecology and Obstetrics (FIGO). Histopathological classification, including the stage, grade and tumor type, was performed by an experienced pathologist (Table 1). The controls included 180 unrelated healthy female volunteers who were matched by age to the cancer patients (Table 1). Written informed consent was obtained from all participating individuals. The procedures of the study were approved by the Local Ethical Committee of Poznań University of Medical Sciences. All women with ovarian cancer and controls were Caucasian from the Wielkopolska area of Poland.

**Table 1:** Clinical characteristics of ovarian cancer patients and healthy controls

Characteristic	Patients (*n* = 159)	Controls (*n* = 180)
Mean age ± SD	55.6 ± 9.6	56.1 ± 8.7
Histological grade		
G1	29 (18.2 %)	
G2	46 (28.9 %)	
G3	44 (27.7 %)	
Gx	40 (25.2 %)	
Clinical stage		
I	44 (27.7 %)	
II	35 (22.0 %)	
III	54 (34.0 %)	
IV	26 (16.3 %)	
Histological type		
Serous	44 (27.7 %)	
Mucinous	27 (17.0 %)	
Endometrioid	47 (29.5 %)	
Clear cell	18 (11.3 %)	
Brenne	3 (1.9 %)	
Mixed	10 (6.3 %)	
Untyped carcinoma	10 (6.3 %)	

### Genotyping

Genomic DNA was isolated from peripheral blood leucocytes by salt extraction. DNA samples were genotyped for the 11 SNPs in *DNMT1*, *DNMT3A* and *DNMT3B* (Supplemental Table 1; Supplemental Fig. 1a–c.). SNPs were selected with the use of the genome browsers of the International HapMap Consortium (http://www.hapmap.org/index.html.en), UCSC (http://genome.ucsc.edu) and dbSNP database (http://www.ncbi.nlm.nih.gov/projects/SNP/). SNPs were selected based on functional significance, location in different LD blocks, and minor allele frequency (MAF) >0.1 in the Caucasian population. Genotyping of the *DNMT1* rs2228611, rs759920, *DNMT3A* rs2289195, rs13401241, rs749131, rs1550117, *DNMT3B* rs2424932 SNPs was performed by high resolution melting curve analysis (HRM) on the LightCycler 480 system (Roche Diagnostics, Mannheim, Germany). Genotyping of the *DNMT1* rs8101626, *DNMT3A* rs7590760, and *DNMT3B* rs1569686, rs2424913SNPs was conducted by PCR, followed by the appropriate restriction enzyme digestion (PCR–RFLP) according to the manufacturer’s instructions (Fermentas, Vilnius, Lithuania). DNA fragments were separated in 2 % agarose gels and visualized by ethidium bromide staining. Primer sequences and conditions for HRM and PCR–RFLP analyses are presented in Supplemental Table 2. Genotyping quality was evaluated by repeated genotyping of 10 % randomly selected samples.

### Statistical analysis

For each SNP, the Hardy–Weinberg equilibrium (HWE) was assessed by Pearson’s goodness-of-fit Chi square (*χ* ^2^) statistic. The differences in the allele and genotype frequencies between cases and controls were determined using standard *χ* ^2^ or Fisher tests. SNPs were tested for association with ovarian cancer using the Cochran-Armitage trend test. The odds ratio (OR) and associated 95 % confidence intervals (95 %CI) were also calculated. The data were analyzed under recessive and dominant inheritance models. To adjust for the multiple testing, we employed a correction factor of 3 (0.005/3 = 0.0167) to take into consideration the number of genes evaluated. This represents a generally accepted correction strategy; one not as stringent as the Bonferroni correction, yet still taking the number of totally independent analyses into account. Pair-wise LD between selected SNPs was computed as both *D*′ and *r* ^2^ values using HaploView 4.0 software (Broad Institute, Cambridge, MA). Haplotype analysis was performed using the UNPHASED 3.1.5 program with the following analysis options: all window sizes, full model and uncertain haplotype option [16]. Haplotypes with a frequency below 0.01 were set to zero. The *p* values for both global and individual tests of haplotype distribution between cases and controls were determined. Statistical significance was assessed using the 1,000-fold permutation test.

High order gene–gene interactions among all tested polymorphic loci were studied by the multifactor dimensionality reduction (MDR) approach (MDR version 2.0 beta 5). A detailed explanation on the MDR method has been described elsewhere [17]. Based on the obtained testing balanced accuracy and cross-validation consistency values, the best statistical gene–gene interaction models were established. A 1,000-fold permutation test was used to assess the statistical significance of MDR models (MDR permutation testing module 0.4.9 alpha).

## Results

### Association of *DNMT1*, *DNMT3A* and *DNMT3B* SNPs with ovarian cancer development

The frequency of all studied genotypes did not exhibit deviation from the HWE between all investigated groups (*p* > 0.05). The lowest (0.08) and highest (0.49) MAF found for the control samples was for *DNMT3A* rs1550117 and *DNMT1* rs2228611 SNPs, respectively (Table 2). The number of genotypes, OR, and 95 % CI calculations for the 11 studied *DNMT1*, *DNMT3B*, and *DNMT3A* SNPs are presented in Table 2. The lowest *p* values of the trend test were observed for the *DNMT1* rs2228611 and rs759920 SNPs in patients with ovarian cancer (*p*_trend_ = 0.0118 and *p*_trend_ = 0.0173, respectively) (Table 2). The statistical significance for multiple testing determined by correction of genes number was *p* = 0.0167. Therefore, we observed, in a recessive inheritance model, that *DNMT1* rs2228611 and rs759920 SNPs are associated with increased risk of ovarian cancer development [OR 1.836 (1.143–2.949), *p* = 0.0114, and OR 1.932 (1.185–3.152), *p* = 0.0078, respectively] (Table 2). However, none of the other nine studied *DNMT1*, *DNMT3B* and *DNMT3A* SNPs exhibited significant association either in dominant or recessive inheritance models with ovarian cancer development (Table 2).

**Table 2:** Association of polymorphisms in genes encoding DNA methyltransferases with the risk of ovarian cancer

Gene	rs no.	Alleles^a^	MAF^b^	Genotypes cases^c^	Genotypes controls^c^	*p*_allelic_ value	*p*_trend_ value	OR_dominant_ (95 % CI)^d^; *p* value	OR_recessive_ (95 % CI)^e^; *p* value
*DNMT1*	rs8101626	A/g	0.44	35/84/40	55/92/33	0.0456	0.0412	1.559 (0.954–2.548); 0.0755	1.497 (0.890–2.520); 0.1272
	rs2228611	A/g	0.49	28/74/57	44/94/42	*0.0117*	*0.0118*	1.514 (0.890–2.575); 0.1247	*1.836* (*1.143–2.949*); *0.0114*
	rs759920	A/g	0.48	32/74/53	46/97/37	0.0179	0.0173	1.362 (0.816–2.274); 0.2359	*1.932* (*1.185–3.152*); *0.0078*
*DNMT3A*	rs2289195	a/G	0.45	48/70/41	61/77/42	0.4226	0.4502	1.185 (0.750–1.874); 0.4667	1.142 (0.696–1.874); 0.6002
	rs7590760	C/g	0.44	62/76/21	53/96/31	0.0728	0.0656	0.653 (0.415–1.026); 0.0638	0.731 (0.401–1.333); 0.3060
	rs13401241	A/c	0.45	55/74/30	56/85/39	0.4110	0.4217	0.854 (0.542–1.345); 0.4956	0.841 (0.494–1.432); 0.5230
	rs749131	G/t	0.45	51/82/26	54/91/35	0.4983	0.4899	0.908 (0.572–1.439); 0.6800	0.810 (0.463–1.417); 0.4595
	rs1550117	a/G	0.08	135/23/1	151/29/0	0.9258	0.9241	0.926 (0.514–1.668); 0.7970	3.416 (0.138–4.529); 0.4690^f^
*DNMT3B*	rs1569686	G/t	0.42	57/73/29	59/90/31	0.7866	0.7878	0.873 (0.557–1.368); 0.5520	1.072 (0.613–1.874); 0.8066
	rs2424913	C/t	0.46	46/86/27	51/91/38	0.5372	0.5259	0.971 (0.606–1.557); 0.9033	0.764 (0.442–1.321); 0.3351
	rs2424932	a/G	0.42	62/64/33	61/88/31	0.8356	0.8414	0.802 (0.515–1.250); 0.3293	1.259 (0.730–2.170); 0.4069

Statistically significant results that persisted after correction for multiple testing are highlighted in italic. Experiment-wide significance threshold required to keep type I error rate at 5 %: 0.0166

^a^Uppercase denotes the more frequent allele in the control samples

^b^ *MAF* minor allele frequency calculated from the control samples

^c^The order of genotypes: DD/Dd/dd (d is the minor allele)

^d^Dominant model: dd + Dd versus DD (d is the minor allele)

^e^Recessive model: dd versus Dd + DD (d is the minor allele)

^f^Fisher exact test

### Association of *DNMT1*, *DNMT3B*, and *DNMT3A* haplotypes with ovarian cancer development

Haplotype analysis of *DNMT1*, *DNMT3B*, and *DNMT3A* polymorphisms did not reveal SNP combinations associated with the risk of ovarian cancer development (Table 3). In patients, the lowest global *p* = 0.0502 was observed for haplotypes composed of the *DNMT1* rs2228611 and rs759920 SNPs (Table 3). However, these results were not statistically significant when permutations were used to generate empiric *p* values. The empirical 5 % quintile of the best *p* value after 1,000 permutations was 0.002566. The selected 11 SNPs situated in distinct regions of *DNMT1*, *DNMT3B*, and *DNMT3A* were either in weak or strong pairwise LD. This was calculated from the control samples, and had *D*′ ranges of 0.934–0.976 for *DNMT1* SNPs, 0.109–1.000 for *DNMT3A* SNPs, and 0.626–0.943 for *DNMT3B* SNPs (Supplementary Table 3).

**Table 3:** Haplotype analysis of the *DNMT1*, *DNMT3A* and *DNMT3B* gene polymorphisms

Gene	SNP combination	*χ* ^2^	Global *p* value
*DNMT1* ^a^	*2-Marker window*		
	rs8101626 rs2228611	5.6892	0.0582
	rs2228611 rs759920	5.9849	0.0502
	*3-Marker window*		
	rs8101626 rs2228611 rs759920	8.3052	0.0810
*DNMT3A*	*2-Marker window*		
	rs2289195 rs7590760	3.3940	0.3348
	rs7590760 rs13401241	2.8982	0.4076
	rs13401241 rs749131	6.5006	0.0896
	rs749131 rs1550117	0.6357	0.8882
	*3-Marker window*		
	rs2289195 rs7590760 rs13401241	4.1816	0.7586
	rs7590760 rs13401241 rs749131	7.1676	0.3056
	rs13401241 rs749131 rs1550117	7.1785	0.2077
	*4-Marker window*		
	rs2289195 rs7590760 rs13401241 rs749131	14.099	0.3669
	rs7590760 rs13401241 rs749131 rs1550117	7.9432	0.4390
	*5-Marker window*		
	rs2289195 rs7590760 rs13401241 rs749131 rs1550117	15.178	0.4387
*DNMT3B*	*2-Marker window*		
	rs1569686 rs2424913	4.0008	0.2614
	rs2424913 rs2424932	1.3364	0.7205
	*3-Marker window*		
	rs1569686 rs2424913 rs2424932	8.7324	0.1202

^a^Empirical 5 % quantile of the best *p* value 0.002566

### MDR analysis of gene–gene interactions among the studied *DNMT1*, *DNMT3A* and *DNMT3B* SNPs

Exhaustive MDR analysis evaluating two- to four-loci combinations of all studied SNPs for each comparison did not reveal statistical significance in predicting susceptibility to ovarian cancer development (Table 4). The best combination of possibly interactive polymorphisms was observed for rs759920 of *DNMT1*, rs2289195 and rs7590760 of *DNMT3A* and rs2424932 of *DNMT3B* (testing balanced accuracy = 59.03 %, cross validation consistency of eight out of ten, permutation test *p* = 0.0940).

**Table 4:** Results of gene–gene interactions analyzed by MDR method

Genes and rs numbers	Testing balanced accuracy	Cross validation consistency (%)	*p* value^a^
*DNMT1*_rs759920, *NMT3A*_rs7590760	0.5866	80	0.1160
*DNMT1*_rs8101626, *DNMT3A*_rs2289195, *DNMT3B*_rs1569686	0.4998	30	0.8530
*DNMT1*_rs759920, *DNMT3A*_rs2289195, *DNMT3A*_rs7590760, *DNMT3B*_rs2424932	0.5903	80	0.0940

^a^Significance of accuracy, empirical *p* value based on 1,000 permutations

## Discussion

As with other cancer types, aberrant DNA methylation encompassing CpG island hypermethylation and global hypomethylation of heterochromatin has been demonstrated in ovarian cancer [4, 5, 12, 18]. To date, it several genes have been found to be down-regulated in ovarian cancer due to their hypermethylation [19–23]. These genes include classical TSGs and genes encoding cell adhesion molecules, proapoptotic proteins, and other proteins supporting ovarian tumorigenesis [19–23]. DNA hypomethylation in ovarian cancer has been found in chromosome 1 satellite 2 and long interspersed nuclear element-1 (LINE-1) repetitive elements [18, 24]. In addition to these findings, many genes are hypomethylated and overexpressed in this type of cancer. Among these are genes encoding activators of the mitogen-activated protein kinases, insulin-like growth factor-2, and proteins associated with chemoresistance [18, 25–27].

Increased levels of some DNMTs have been observed in ovarian cancer cell lines and primary ovarian cancerous tissues as compared to normal ovarian cells [10, 11, 28]. Ahluwalia et al. [10] found significantly higher DNMT1 and DNMT3B mRNA levels in ovarian cancer cells than in normal ovarian surface epithelial cells. Moreover, Chen et al. [28] observed significantly elevated DNMT1 and DNMT3B mRNA levels in primary and recurrent epithelial ovarian carcinoma compared to normal epithelial ovarian cells. Recently, Cheng et al. (2011) demonstrated that DNMT1, but not DNMT3A or DNMT3B, levels were increased in serous borderline ovarian tumor cells. They also demonstrated that DNMT1 levels correlated with E-cadherin promoter methylation [11].

In our study we found that the *DNMT1* rs2228611 and rs759920 SNPs can be risk factors of ovarian cancer in a Polish population. To date, the *DNMT1* rs2228611 SNP has been associated with sporadic infiltrating ductal breast carcinoma among Chinese Han women in the Heilongjiang Province [29]. Moreover, the *DNMT1* rs2228611 SNP modified associations between urinary cadmium and hypomethylation of LINE-1 repeated sequences [30]. In addition to these findings, the two intronic polymorphisms rs2241531 and rs4804490 of *DNMT1* may be associated with hepatitis B virus (HBV) clearance and protection from the development of hepatocellular carcinoma [31].

Our studies did not demonstrate an association of the *DNMT3B* rs1569686, rs2424913 and rs2424932 SNPs with ovarian cancer. To date, the *DNMT3B* rs2424913 polymorphism has been demonstrated to be a risk factor of lung, breast, and head and neck cancers and age of onset of hereditary nonpolyposis colorectal cancer (CRC) [32–35]. Furthermore, the *DNMT3B* rs2424913 SNP may contribute to reduced methylation of CpG islands of the *MYOD* and *MLH1* genes in normal colonic mucosa of patients with CRC, and with increased promoter methylation of TSGs related to the development of prostate cancer [36, 37]. Another polymorphism of *DNMT3B*, rs2424909, being in great LD with rs2424913 has been found to be a genetic risk factor of gastric cancer, CRCs, and gallbladder carcinoma [38–40].

It has recently been found that the *DNMT3A* polymorphism rs1550117 alters the promoter activity and risk of gastric cancer and CRC [14, 15]. However, we did not find an association of any of the five studied *DNMT3A* SNPs with ovarian cancer development. Frequent mutations in *DNMT3A* have been demonstrated in human T cell lymphoma, myelodysplastic syndromes, as well as myeloid and monocytic leukemias [41–44]. Moreover, the *DNMT3A* intronic polymorphism rs13428812, situated in the same LD block with our studied rs7590760, has been shown to contribute to Crohn’s disease [45].

DNMT3A and DNMT3B carry out de novo methylation [7, 13]. DNMT3A and DNMT3B are involved in imprinting of germ cells and normal embryonic development [46, 47]. Moreover, DNMT3A contributes to the differentiation of neural progenitors and hematopoietic stem cells [48, 49]. DNMT1 is responsible for the maintenance of the hemimethylated DNA methylation pattern during DNA replication, and also has a role in gene silencing [13, 50].

Our study may suggest that Polish women bearing either the *DNMT1* rs2228611 or rs759920 SNP may have an increased risk of ovarian cancer. Neither of these polymorphisms is a functional variant, and their association with ovarian cancer may be due to LD with one or more functional polymorphisms of *DNMT1*. Therefore, to confirm the role of these SNPs in ovarian cancer, this study should be repeated in a larger and independent cohort, and functional studies of these SNPs must be conducted. Moreover, a future genome-wide SNPs genotyping study in our sample collection will additionally provide more knowledge of the genes contributing to the onset of this disease.

## Electronic supplementary material

Below is the link to the electronic supplementary material.

(JPG 2922 kb)

(JPG 4199 kb)

(JPG 4757 kb)

Fig. 1 The linkage disequilibrium (LD) plot of HapMap SNPs within the *DNMT1* (a), *DNMT3A* (b) and *DNMT3B* (c) regions. The plot was generated using the genotype data from HapMap CEU samples and the Haploview 4.0 software (Broad Institute, Cambridge, MA). The names of the examined SNPs are enclosed in boxes. The numbers in the squares indicate percentage of LD between a given pair of SNPs (*D*′ values). (DOC 21 kb)

Table 1 Characteristics of polymorphisms genotyped in the data set (DOC 35 kb)

Table 2 HRM and RFLP conditions for the identification of polymorphisms genotyped in the data set (DOC 56 kb)

Table 3 Linkage disequilibrium between markers of the *DNMT1* gene in the control samples. Linkage disequilibrium between markers of the *DNMT3A* gene in the control samples (DOC 37 kb)
