# Supplementary figures and images for: DNMT1, DNMT3A and DNMT3B gene variants in relation to ovarian cancer risk in the Polish population
Source: Mol Biol Rep. 2013 May 12;40(8):4893–9. doi: 10.1007/s11033-013-2589-0 (PMC3723978; doi:10.1007/s11033-013-2589-0)

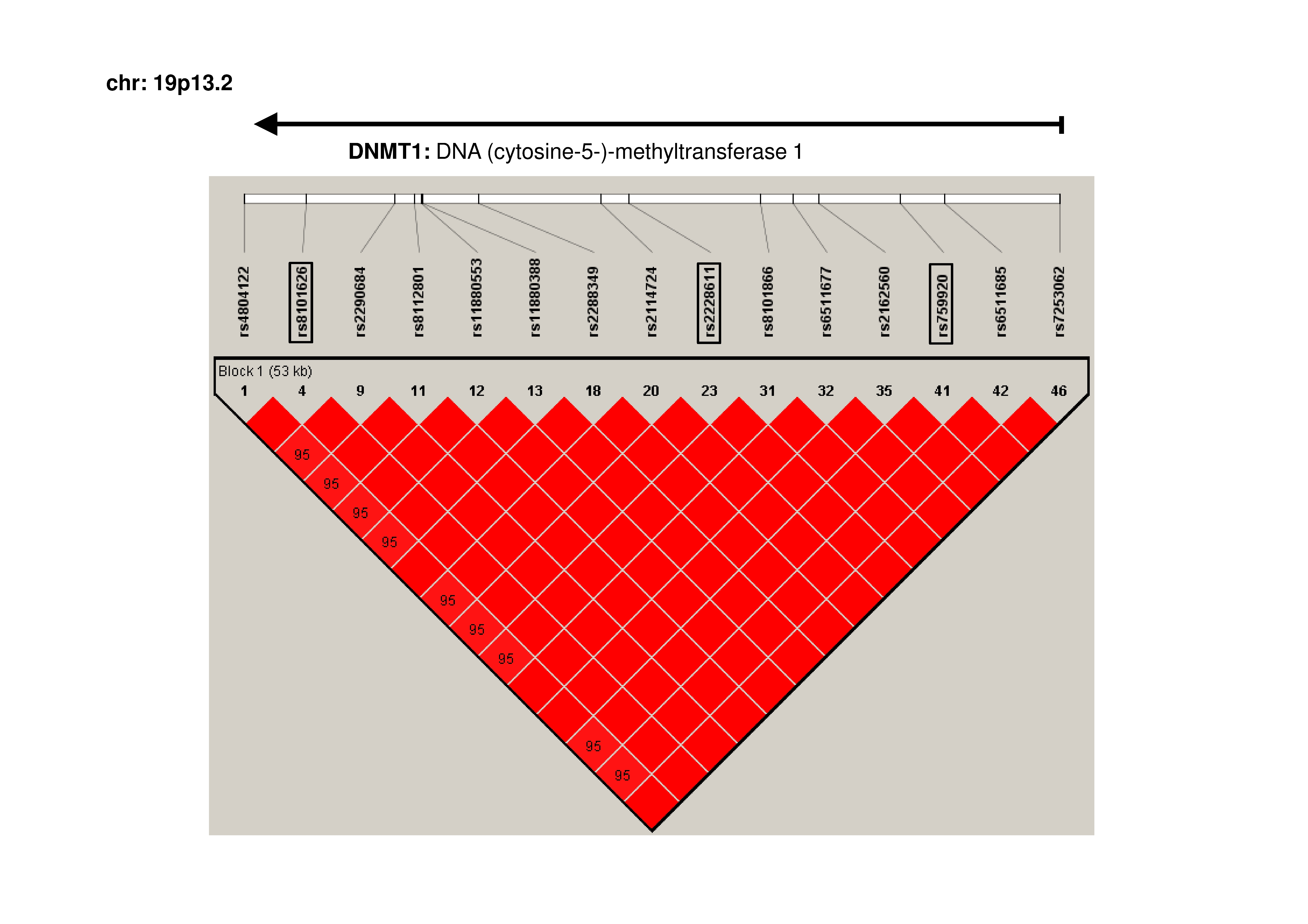

Supplement: Supplementary file 1 — (JPG 2922 kb) [file 11033_2013_2589_MOESM1_ESM.jpg]

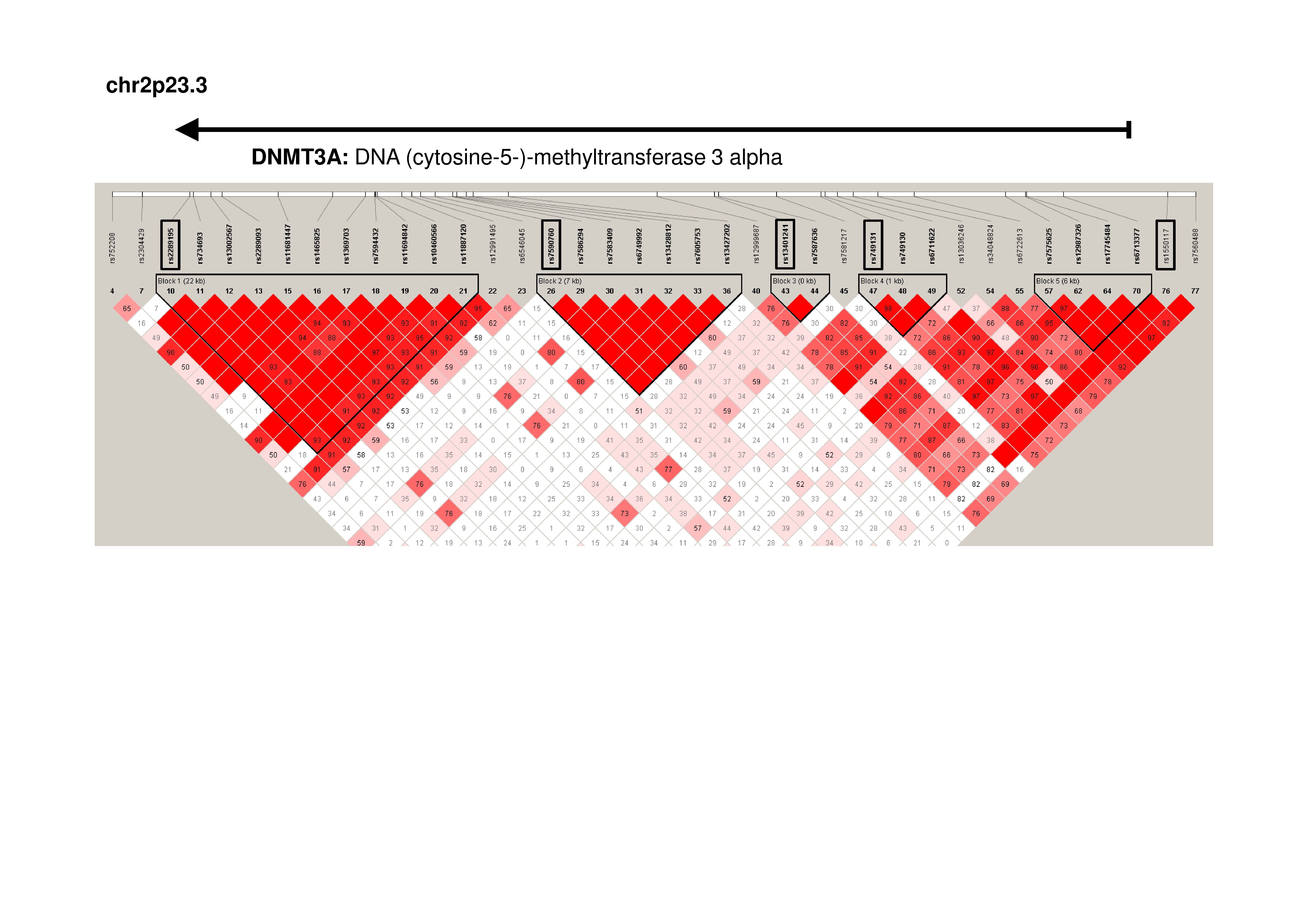

Supplement: Supplementary file 2 — (JPG 4199 kb) [file 11033_2013_2589_MOESM2_ESM.jpg]

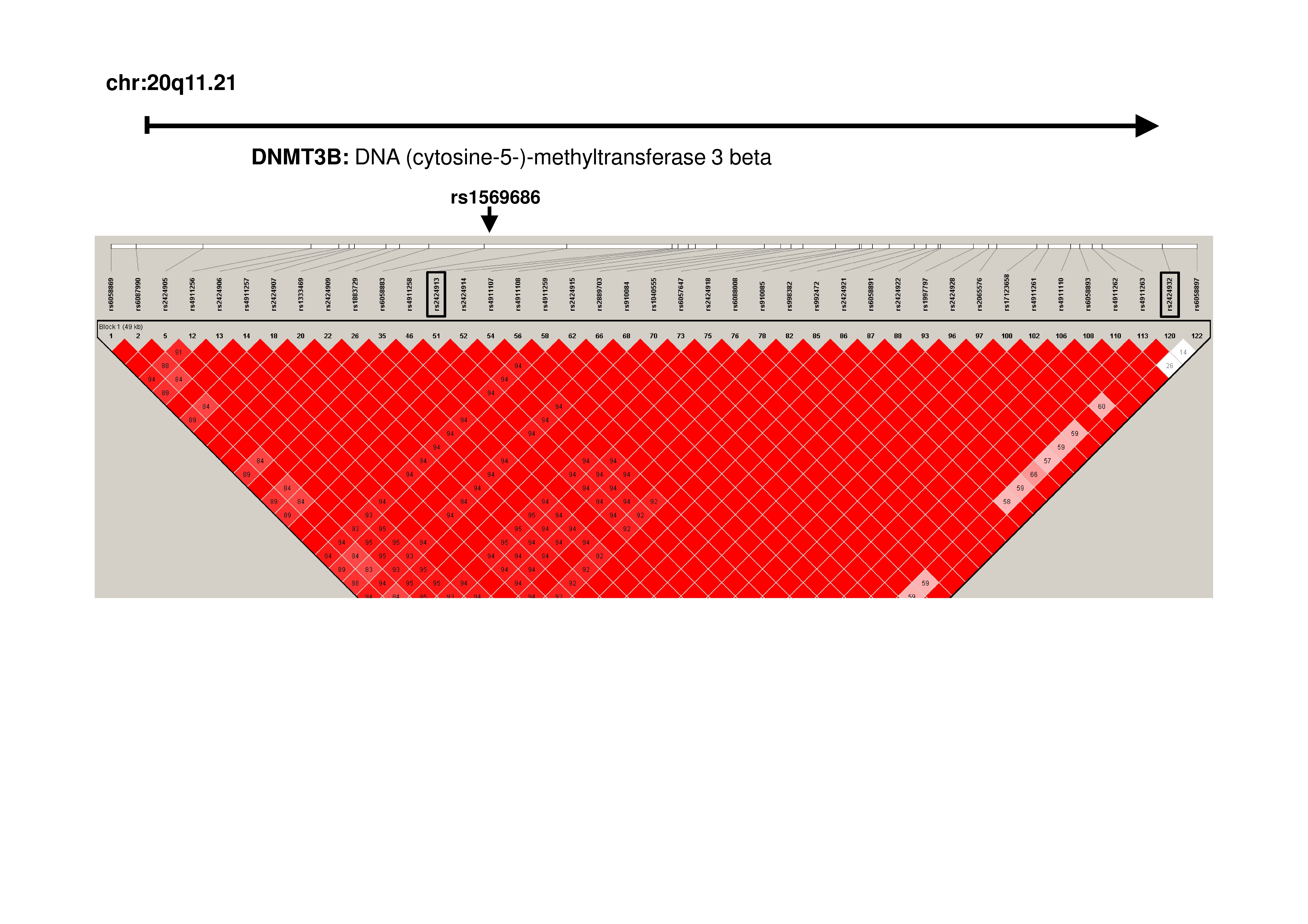

Supplement: Supplementary file 3 — (JPG 4757 kb) [file 11033_2013_2589_MOESM3_ESM.jpg]
