# Supplementary material for: DNMT1, DNMT3A and DNMT3B gene variants in relation to ovarian cancer risk in the Polish population
Source: Mol Biol Rep. 2013 May 12;40(8):4893–9. doi: 10.1007/s11033-013-2589-0 (PMC3723978; doi:10.1007/s11033-013-2589-0)
Supplement: Supplementary file 4 — The linkage disequilibrium (LD) plot of HapMap SNPs within the DNMT1 (a), DNMT3A (b) and DNMT3B (c) regions. The plot was generated using the genotype data from HapMap CEU samples and the Haploview 4.0 software (Broad Institute, Cambridge, MA). The names of the examined SNPs are enclosed in boxes. The numbers in the squares indicate percentage of LD between a given pair of SNPs (D′ values). (DOC 21 kb) [file 11033_2013_2589_MOESM4_ESM.doc]

**Figure legends**

**Supplemental Fig. 1. The Linkage Disequilibrium (LD) plot of HapMap SNPs within the *DNMT1* (A)*, DNMT3A* (B) *and DNMT3B* (C) regions.** The plot was generated using the genotype data from HapMap CEU samples and the Haploview 4.0 software (Broad Institute, Cambridge, MA). The names of the examined SNPs are enclosed in boxes. The numbers in the squares indicate percentage of LD between a given pair of SNPs (D’ values).
