# Supplementary material for: DNMT1, DNMT3A and DNMT3B gene variants in relation to ovarian cancer risk in the Polish population
Source: Mol Biol Rep. 2013 May 12;40(8):4893–9. doi: 10.1007/s11033-013-2589-0 (PMC3723978; doi:10.1007/s11033-013-2589-0)
Supplement: Supplementary file 5 — Characteristics of polymorphisms genotyped in the data set (DOC 35 kb) [file 11033_2013_2589_MOESM5_ESM.doc]

**Supplemental Table 1. Characteristics of polymorphisms genotyped in the data set**.

| **Gene symbol** | **Gene name** | **Location** | **rs no.** | **SNP location^a^** | **SNP function^b^** | **Alleles^c^** | **MAF^d^** |
| --- | --- | --- | --- | --- | --- | --- | --- |
| ***DNMT1*** | DNA (cytosine-5-)-methyltransferase 1 | 19p13.2 | rs8101626 | chr19:10246029 | intronic | A/g | 0.44 |
|  |  |  | rs2228611 | chr19:10267077 | cds-synon (Pro447Pro) | A/g | 0.49 |
|  |  |  | rs759920 | chr19:10284778 | intronic | A/g | 0.48 |
| ***DNMT3A*** | DNA (cytosine-5-)-methyltransferase 3 alpha | 2p23.3 | rs2289195 | chr2:25463483 | intronic | a/G | 0.45 |
|  |  |  | rs7590760 | chr2:25489183 | intronic | C/g | 0.44 |
|  |  |  | rs13401241 | chr2:25518470 | intronic | A/c | 0.45 |
|  |  |  | rs749131 | chr2:25529624 | intronic | G/t | 0.45 |
|  |  |  | rs1550117 | chr2:25565907 | nearGene-5 | a/G | 0.08 |
| ***DNMT3B*** | DNA (cytosine-5-)-methyltransferase 3 beta | 20q11.21 | rs1569686 | chr20:31367079 | intronic | G/t | 0.42 |
|  |  |  | rs2424913 | chr20:31374259 | intronic | C/t | 0.46 |
|  |  |  | rs2424932 | chr20:31396536 | UTR-3 | a/G | 0.42 |

^a^Based on UCSC Human Genome Browser, February 2009 human reference sequence (GRCh37).

^b^According to the Single Nucleotide Polymorphism database (dbSNP).

^c^Uppercase denotes the more frequent allele in the control samples.

^d^MAF, minor allele frequency calculated from the control samples.
