# Supplementary material for: DNMT1, DNMT3A and DNMT3B gene variants in relation to ovarian cancer risk in the Polish population
Source: Mol Biol Rep. 2013 May 12;40(8):4893–9. doi: 10.1007/s11033-013-2589-0 (PMC3723978; doi:10.1007/s11033-013-2589-0)
Supplement: Supplementary file 6 — HRM and RFLP conditions for the identification of polymorphisms genotyped in the data set (DOC 56 kb) [file 11033_2013_2589_MOESM7_ESM.doc]

**Supplemental Table 2**. HRM and RFLP conditions for the identification of polymorphisms genotyped in the data set.

|  |  |  |  |  |  |  | **HRM analysis^c^** | **RFLP analysis^d^** | |
| --- | --- | --- | --- | --- | --- | --- | --- | --- | --- |
| **Gene symbol** | **rs no.** | **Location^a^** | **Alleles^b^** | **Primers for PCR amplification** | **Annealing temp. (°C)** | **PCR product length (bp)** | **Melting temp. range (°C)** | **Restriction enzyme** | **Restriction fragment length (bp)** |
|  |  |  |  | **(5’ – 3’)** |  |  |  |  |  |
| ***DNMT1*** | **rs8101626** | chr19:10246029 | A/g | F: CAAATGGGCCACCTAGACAC | 67.0°C | 640 bp |  | Alw26I | allele A: 640 |
|  |  |  |  | R: GGCAGAGATTGAGCCAGAAG |  |  |  |  | allele G: 474 + 166 |
|  | **rs2228611** | chr19:10267077 | A/g | F: AGTGTGCCCCAAACATAATCC | 60.6°C | 179 bp | 80 - 90°C |  |  |
|  |  |  |  | R: GCATGTGCTTTGTTTCCTGTC |  |  |  |  |  |
|  | **rs759920** | chr19:10284778 | A/g | F: ATTTGACATGGTTAGAGCAAGAGTG | 60.6°C | 136 bp | 80 - 90°C |  |  |
|  |  |  |  | R: AGTGTGCCTGCCTACTTCTCG |  |  |  |  |  |
| ***DNMT3A*** | **rs2289195** | chr2:25463483 | a/G | F: CCAAGGAGGAAGCCTATGTG | 60.6°C | 113 bp | 82 – 89°C |  |  |
|  |  |  |  | R: CCTGCAATGACCTCTCCATC |  |  |  |  |  |
|  | **rs7590760** | chr2:25489183 | C/g | F: TGCTGTGCCTACTCCAAACA | 62.6°C | 343 bp |  | Csp6I | allele C: 267 + 76 |
|  |  |  |  | R: GCCATGAATGTCCAGAAGGT |  |  |  |  | allele G: 343 |
|  | **rs13401241** | chr2:25518470 | A/c | F:CCACACACTTTTTCCAGCAG | 60.6°C | 129 bp | 79 – 86°C |  |  |
|  |  |  |  | R: CCAACTGCAAAGCCTATGGT |  |  |  |  |  |
|  | **rs749131** | chr2:25529624 | G/t | F: ATGTCACAAGGCCAGACACA | 60.6°C | 140 bp | 78 – 85°C |  |  |
|  |  |  |  | R: TCAGCATGGCATCAGGTACT |  |  |  |  |  |
|  | **rs1550117** | chr2:25565907 | a/G | F: GGGCTCTAGGTTCCTGACTTG | 60.6°C | 83 bp | 78 - 87°C |  |  |
|  |  |  |  | R: CTGGAGGAGTGAGATGAGCAC |  |  |  |  |  |
| ***DNMT3B*** | **rs1569686** | chr20:31367079 | G/t | F: GACCTGGAGCTGTTTGTGGT | 67.0°C | 550 bp |  | PvuII | allele G: 550 |
|  |  |  |  | R: CAGAGTCCCAAGTTCCCAAG |  |  |  |  | allele T: 420 + 130 |
|  | **rs2424913** | chr20:31374259 | C/t | F: TTGTCCTGAAGCTGGCTACC | 66.3°C | 431 bp |  | AvrII | allele C: 431 |
|  |  |  |  | R: ACCAGGAGAGAAGCCAACAG |  |  |  |  | allele T: 362 + 69 |
|  | **rs2424932** | chr20:31396536 | a/G | F: AAGGGGTGTGCTGAGTTCTA | 60.6°C | 108 bp | 76 - 86°C |  |  |
|  |  |  |  | R: AAGCTCCTTGCTTCACACTC |  |  |  |  |  |

^a^Based on UCSC Human Genome Browser, February 2009 human reference sequence (GRCh37).

^b^Uppercase denotes the more frequent allele in the control samples.

^c^HRM analysis, High Resolution Melt analysis.

^d^RFLP analysis, Restriction Fragment Length Polymorphism analysis.
