# Supplementary material for: DNMT1, DNMT3A and DNMT3B gene variants in relation to ovarian cancer risk in the Polish population
Source: Mol Biol Rep. 2013 May 12;40(8):4893–9. doi: 10.1007/s11033-013-2589-0 (PMC3723978; doi:10.1007/s11033-013-2589-0)
Supplement: Supplementary file 7 — Linkage disequilibrium between markers of the DNMT1 gene in the control samples. Linkage disequilibrium between markers of the DNMT3A gene in the control samples (DOC 37 kb) [file 11033_2013_2589_MOESM6_ESM.doc]

**Supplemantal Table 3.**

**Linkage disequilibrium between markers of the *DNMT1* gene in the control samples.**

|  | **rs8101626** | **rs2228611** | **rs759920** |
| --- | --- | --- | --- |
| **rs8101626** |  | 0.958 | 0.934 |
| **rs2228611** | 0.724 |  | 0.976 |
| **rs759920** | 0.744 | 0.891 |  |

**Linkage disequilibrium between markers of the *DNMT3A* gene in the control samples.**

|  | **rs2289195** | **rs7590760** | **rs13401241** | **rs749131** | **rs1550117** |
| --- | --- | --- | --- | --- | --- |
| **rs2289195** |  | 0.159 | 0.109 | 0.168 | 0.289 |
| **rs7590760** | 0.016 |  | 0.289 | 0.298 | 1.000 |
| **rs13401241** | 0.012 | 0.079 |  | 0.857 | 0.449 |
| **rs749131** | 0.027 | 0.086 | 0.717 |  | 0.540 |
| **rs1550117** | 0.006 | 0.113 | 0.021 | 0.032 |  |

**Linkage disequilibrium between markers of the *DNMT3B* gene in the control samples.**

|  | **rs1569686** | **rs2424913** | **rs2424932** |
| --- | --- | --- | --- |
| **rs1569686** |  | 0.880 | 0.943 |
| **rs2424913** | 0.661 |  | 0.626 |
| **rs2424932** | 0.476 | 0.244 |  |
